# Perceived Closeness and Autistic Traits Modulate Interpersonal Vocal Communication

**DOI:** 10.3389/fpsyt.2020.00050

**Published:** 2020-02-28

**Authors:** T. A. Sumathi, Olivia Spinola, Nandini Chatterjee Singh, Bhismadev Chakrabarti

**Affiliations:** ^1^ National Brain Research Centre, Language, Literacy and Music Laboratory, Manesar, India; ^2^ Department of Psychology, Universita` degli Studi di Milano Bicocca, Milan, Italy; ^3^ Centre for Autism, School of Psychology & Clinical Language Sciences, University of Reading, Reading, United Kingdom; ^4^ Department of Psychology, Sapienza University of Rome, Rome, Italy; ^5^ Inter University Centre for Biomedical Research, Mahatma Gandhi University, Kottayam, India; ^6^ India Autism Center, Kolkata, India

**Keywords:** interpersonal closeness, dyad, vocal modulation, autism, social behavior

## Abstract

Vocal modulation is a critical component of interpersonal communication. It not only serves as a dynamic and flexible tool for self-expression and linguistic information but also plays a key role in social behavior. Variation in vocal modulation can be driven by individual traits of interlocutors as well as factors relating to the dyad, such as the perceived closeness between interlocutors. In this study we examine both of these sources of variation. At an individual level, we examine the impact of autistic traits, since lack of appropriate vocal modulation has often been associated with Autism Spectrum Disorders. At a dyadic level, we examine the role of perceived closeness between interlocutors on vocal modulation. The study was conducted in three separate samples from India, Italy, and the UK. Articulatory features were extracted from recorded conversations between a total of 85 same-sex pairs of participants, and the articulation space calculated. A larger articulation space corresponds to greater number of spectro-temporal modulations (articulatory variations) sampled by the speaker. Articulation space showed a positive association with interpersonal closeness and a weak negative association with autistic traits. This study thus provides novel insights into individual and dyadic variation that can influence interpersonal vocal communication.

## Introduction

The human voice is unique in its repertoire and functional utility. In addition to its role in expressing oneself through linguistic and non-linguistic routes, it acts as a crucial tool for social communication from early in development (1). Humans routinely and volitionally modulate their voice in social contexts like striking up a new friendship, arguing or making an emotionally charged speech. A well-modulated voice carries considerable information about the message, speaker, language, and even on the emotions of the speaker (2–4). The dynamic nature of voice is vital for its social function, as vocal modulation can affect both the speaker and the addressee (5). Impairments in the recognition of socially communicative vocal modulation, such as emotional prosody, have been associated with impaired psychosocial functioning, as seen in autism or schizophrenia (6).

Several factors have been found to influence context-specific vocal modulation. In acoustic terms, voice modulation is defined as the manipulation of any non-verbal property of the voice including, but not limited to, pitch (F0) and formant frequencies. A recent account has distinguished two different types of vocal modulations. One of these types is involuntary, elicited automatically by environmental stimuli or different levels of endogenous arousal, and the other is a more controlled vocal modulation, which is goal-directed and less dependent on external stimuli, though not necessarily voluntary (1).

In its most basic form, the properties of the voice modulated speech signal are constrained by the physical and physiological properties of the speech production apparatus such as the thickness and characteristics of the vocal folds, variance in the shape and dimensions of a person's palate, and the dynamic use of the vocal tract (7). Thus, some acoustic variability can be attributed to the physical constraints and capabilities of the speaker, which vary with age, gender, and hormonal factors (8). Apart from this, speakers vary greatly in their articulatory habits and speaking style, both of which are functions of linguistic background, emotion-related state and trait measures of the speaker, acoustic environment, as well as the social context (7).

The social context-dependency of controlled vocal modulation remains relatively under researched. People communicate differently depending on their type of relationship, e.g., the way a person talks to friends is different from the way s/he talks to a stranger, or to a pet, or to a police officer (7). Consistent with this heuristic, Pisanski and others reported pitch modulation to be predictive of mate choice behavior (8). In another study by the same group, human males were shown to be able to volitionally exaggerate their body size by suitably modulating specific acoustic parameters of their voice (9). The role of social context and the relevance of relationship closeness in acoustic vocal modulation were studied by Katerenchuk and colleagues by extracting low-level acoustic features from a corpus of native English speech telephone conversations, which were then used to distinguish a conversation between friends or members of a family (10). Their results indicated that it is possible to distinguish friends from family on the basis of some low level lexical and acoustic signals. Similar results were reported from another study which examined the speech of a single Japanese speaker as she spoke to different conversational partners, finding significant differences in pitch (F0) and normalized amplitude quotient (NAQ) depending on the relationship between the speaker and her conversational partner (11). These preliminary studies provide important evidence on how voice modulation can be affected by context-specific factors such as dyadic relationship. However, “friend pairs” or “family pairs” are somewhat arbitrary categories, within which the relationship between the interlocutors can vary greatly, depending on the individuals involved. To get around this variability, an alternative approach involves asking each member of a conversing pair to rate the perceived closeness toward the other member of the pair. This dimensional approach to evaluate the dyadic relationship is akin to the widely-used metric of “social distance” (12). Social distance in this sense, relates more to subjective closeness (perception of relationship quality) rather than to degree and variety of social interaction (13).

Modulation of vocal communication can also be influenced by individual-level factors, and not just on those specific to the dyad. Autism-related traits might constitute one such dimension of individual variability. Anecdotal reports have suggested an “autistic monotone” in describing an absence of context-appropriate vocal modulation in interpersonal communication in individuals with Autism Spectrum Disorders (ASD) (14). However, this suggested feature is not driven by group differences in pitch range, since case-control studies of pitch profiles in children with and without ASD have shown equivalent or larger pitch ranges in ASD (15). We were also interested to examine the impact of autistic traits on vocal modulation, given its critical role in context-appropriate social communication an area associated with deficits in individuals with ASD. Autistic traits exist across a continuum across the population (16, 17) and measuring individual autistic traits allows for a direct test of their impact on vocal modulation.

Having described the two sources of variability of interest to the current study, we describe below our methodological operationalization of the key term “vocal modulation,” i.e. change of voice over time in intensity, and frequency.

Past studies of voice modulation have used features like voice onset and offset times (VOT) (18) or voiced pitch (8). Studies of voice modulation not only involve tedious procedures of acoustic analysis, but also primarily examine speech features associated with specific time scales, isolated from one another. For instance, VOT studies primarily focus on production of stops and fricatives at time scales of 10 to 20 milliseconds while studies of voiced pitch and formant transitions investigate spectral changes around 30 to 50 milliseconds. However, the speech signal is not characterized by isolated spectrotemporal events but instead by joint spectro-temporal events that occur over multiple time windows and many frequency bands. These patterns carry important spectro-temporal information regarding both linguistic and non-linguistic features of speech as a whole (19). The Speech Modulation Spectrum (SMS) was developed to quantify the energy in various temporal and spectral modulations, by calculating the two-dimensional (2D) Fourier transform of the spectrogram (20). Specifically the SMS is focused on characterizing the spectro-temporal power in three articulatory features of speech which are at different time scales, namely syllabicity that is buried in temporal events at hundreds of milliseconds, formant transitions encoded around 25 to 40 milliseconds and place of articulation around 10 to 20 milliseconds (21, 22).

The collection of these different articulatory gestures in a voice signal are represented in an “articulation space,” and it provides a spectro-temporal energy distribution of different articulatory gestures The area of the “articulation space” has been used to compare individual differences in vocal modulation by comparing speech imitation abilities (23). The results of this study showed that individuals with high abilities for speech imitation had an expanded “articulation space,” which allowed them access to a larger repertoire of sounds thereby providing them with greater flexibility in pronunciation. In another study on the emergence of articulatory gestures in early development, significant correlations were found between the area occupied by different articulatory gestures and language and motor ability as assessed by the Mullen and the Vineland scales in toddlers with ASD (24). Accordingly, the area of the articulation space was chosen as an index of vocal modulation in the current study.

In light of anecdotal reports and previous related studies, we hypothesized that higher closeness rating for the listener will be associated with an increased number of articulatory gestures by the speaker (and hence greater articulation space). We also hypothesized that individuals high in autistic traits would exhibit reduced articulation space, indexing a reduced use of articulatory gestures.

## Methods

All protocols were approved by the Research Ethics Committees of the University of Reading, Università degli studi di Milano Bicocca, and National Brain Research Centre India.

### Participants

170 healthy volunteers from three countries (43 native English speaker pairs of participants (18 male pairs, 25 female pairs) from UK, 22 pairs (9 male pairs, 13 female pairs) from India and 20 pairs (10 male pairs, 10 female pairs) from Italy participated in this study.

### Procedure

Participants were asked to come along with another same-gender person, who could be either a friend or an acquaintance. Each participant was asked to rate his/her perceived closeness to the other member of the pair through a Closeness Rating scale on a 10-point Likert scale (where closeness rating of 0 indicated very low closeness and 10 indicated high closeness), similar to previous studies (12, 25). Participants were not allowed to see each other's Closeness Rating. All participants were also asked to fill in the Autism Spectrum Quotient (26). The original questionnaire was provided to India and UK participants, and the Italian translated version (27) was administered to participants in Italy. Participants were required to sit in front of each other in a silent room with a voice recording device in the middle. Once participants were comfortably seated, each participant was given one of two colored images (abstract paintings) printed on paper and was asked to describe the image to the other participant in as much detail as possible for around 2:30 minutes each. The experimenter then left the room. Each pair was instructed to speak one by one, to minimize the impact of overlapping voices and background noises. Participants' speech was recorded by a ZOOM H1 Handy recorder in India, and an iPhone in UK and in Italy. The distance of the recorder was kept constant for all recordings. Since English is the primary language for official communication in UK as well as India, participants in these two countries spoke in English. All participants in the Italian sample spoke in Italian.

### Analysis

All participants' speech was manually heard and cleaned using Goldwave (version 5.69) and resampled as PCM signed 16 bit mono, 22,050 Hz sampling rate in WAV format. The speech data was edited manually and non-speech utterances such as laughs, cough, etc. were removed. The amplitude of the waveforms was normalized to −18 db for all speech data. Speech Modulation Spectra were calculated for each participant using custom developed code developed in MATLAB R2011a (28). Statistical analysis was carried out using SPSS (v14) and jamovi (29). Finally, articulation space, length of the cleaned speech recordings (articulation time), closeness rating, and AQ measures were used for further analysis in all three data sets.

**Figure 1:**
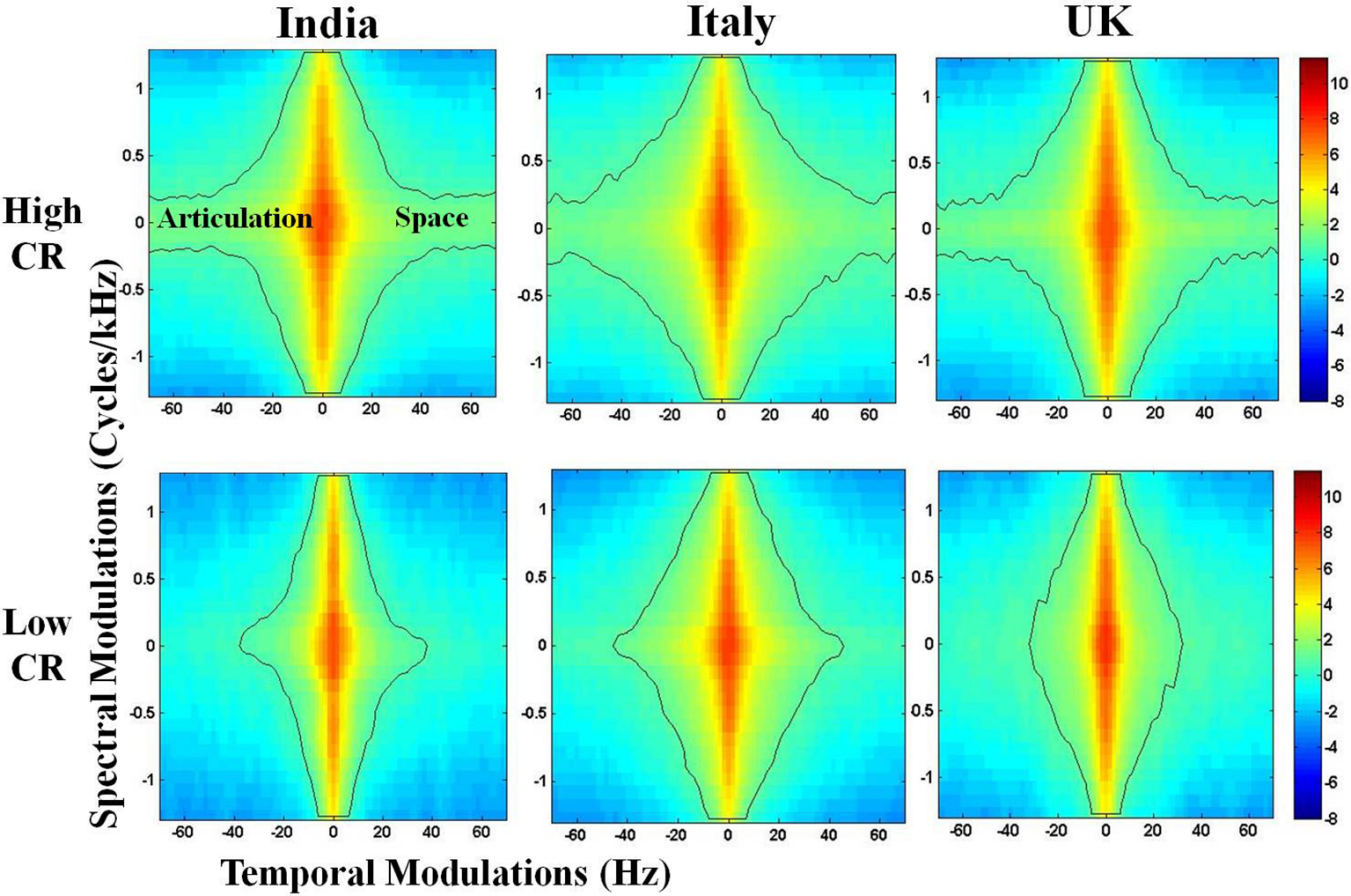
Representative speech modulation spectra for individuals rated high and low on closeness rating (CR) from three countries. Articulation Space is quantified through the number of pixels enclosed within the contour, and encompasses 99.9% of the energy in the distribution of spectro-temporal modulations. Color-coded bar reflects the intensity of energy/power distribution. Note the differences in articulation space between high CR and low CR.

The first step of this analysis involves using speech samples from each participant to calculate a spectrogram. The spectrogram is a time–frequency representation of the speech signal and offers a visual display of fluctuations in frequency and time, described respectively as spectral and temporal modulations. As described earlier, the speech modulation spectrum is obtained by estimating the 2-D Fourier decomposition of the spectrogram, which yields a probability distribution of these spectral and temporal modulations (28). Earlier studies in speech perception (22, 30) have shown spectral and temporal modulations at different time scales encode different aspects of speech. In a typical SMS (20), the central region between 2 and 10 Hz carries supra-segmental information and encodes syllabic rhythm. The side lobes between 10 and 100 Hz carry information about segmental features. Formant transitions are encoded between 10 and 40 Hz, and place of articulation information is found between 40 and 100 Hz (28). As the SMS goes from 1 to 100 Hz, the amplitude fluctuations of a sound become faster and go from syllabic to vowel-like to plosive-like segments (21). The SMS thus plots an “articulation space” which depicts how energy or “power” is distributed in different articulatory features of spoken language, namely syllabic rhythm, formant transitions, and place of articulation (**Figure 1**). The SMS was plotted for each participant, and the contour area (hereafter referred to as “articulation space”) was estimated by counting the total number of pixels within the contour which covers 99% energy of the speech from 1 to 100 Hz (20, 23). The contour area from 1 to 100 Hz corresponds to all the articulatory features mentioned above (detailed description in (20)). Previous studies used this method and demonstrated its construct validity by testing its correlation with other behavioral measures, such as speech motor functions (23, 24).

A hierarchical linear model (HLM) was constructed with articulation space as the predicted variable, and the following predictors as fixed factors (gender, country, age, duration, CR, AQ). Participants were nested in pairs, with random intercepts for each pair. Restricted maximum likelihood was used to estimate the model parameters.

## Results

### Data Descriptives

Participant ages ranged from 18 to 33 years across all three samples (Italy, 19–29; India, 19–33; UK, 18–23). AQ scores ranged from 5 to 40 across all three samples (Italy, 7–31; India, 15–40; UK, 5–31). AQ scores were not available for four pairs from the India data set and one pair from the UK data set. Closeness ratings ranged from 0 to 10 across all three samples (Italy, 1–9; India, 1–10; UK, 0–10). The score ranges in all three samples are comparable to previous studies using these measures done in neurotypical young adult samples.

Descriptive statistics on all key measures for each sample, split by gender, are provided in **Table 1**.

**Table 1:** Data descriptives.

		INDIA			ITALY			UK		
		N	M	SD	N	M	SD	N	M	SD
**CR**	**Male**	18	5.2	3.1	20	7.0	2.2	36	8.1	1.6
	**Female**	26	4.5	2.5	20	5.7	2.7	50	3.9	3.5
	**Total**	44	4.4	2.6	40	6.3	2.5	86	5.7	3.5
**AQ**	**Male**	16	23.3	5.3	20	17.7	6.7	36	14.8	3.9
	**Female**	20	22.5	5.8	20	13.2	4.4	48	16.3	5.0
	**Total**	36	22.8	5.2	40	15.5	6.0	84	15.6	4.6
**Articulation space**	**Male**	18	208.8	44.2	20	314.0	83.5	36	326.2	103.4
	**Female**	26	210.6	33.4	20	233.5	36.0	50	239.8	49.4
	**Total**	44	209.9	37.7	40	273.8	75.5	86	276.0	87.4

CR, closeness rating; AQ, autism spectrum quotient.

The HLM analysis revealed a significant effect of CR on articulation space (*t* = 2.82; *p* < 0.01; 95% CI [1.71–9.5]). The effect of AQ on articulation space fell below the traditional threshold of statistical significance (*t* = −1.73; *p* = 0.085; 95% CI [−3.9 to 0.24]) (**Table 2**).

**Table 2:** Results of the Hierarchical Linear Model-based Analysis.

				95% Confidence Interval				
Names	Effect	Estimate	SE	Lower	Upper	df	t	p
(Intercept)	(Intercept)	255.655	6.437	243.037	268.272	21.4	39.714	<.001
Gender 1	2–1	51.015	12.082	27.336	74.694	113.1	4.223	<.001
Country 1	India–Italy	−47.394	18.082	−82.833	−11.954	141.8	−2.621	0.010
Country 2	UK–Italy	13.184	15.324	−16.850	43.219	151.8	0.860	0.391
Age	Age	2.507	2.428	−2.252	7.266	145.1	1.033	0.304
Duration	Duration	−0.168	0.231	−0.621	0.284	139.8	−0.729	0.467
CR	CR	5.603	1.989	1.705	9.501	147.5	2.818	0.006
AQ	AQ	−1.834	1.056	−3.904	0.236	150.0	−1.736	0.085

CR, closeness rating; AQ, autism spectrum quotient; SE, standard error; gender 1, female; gender 2, male.

**Figure 2:**
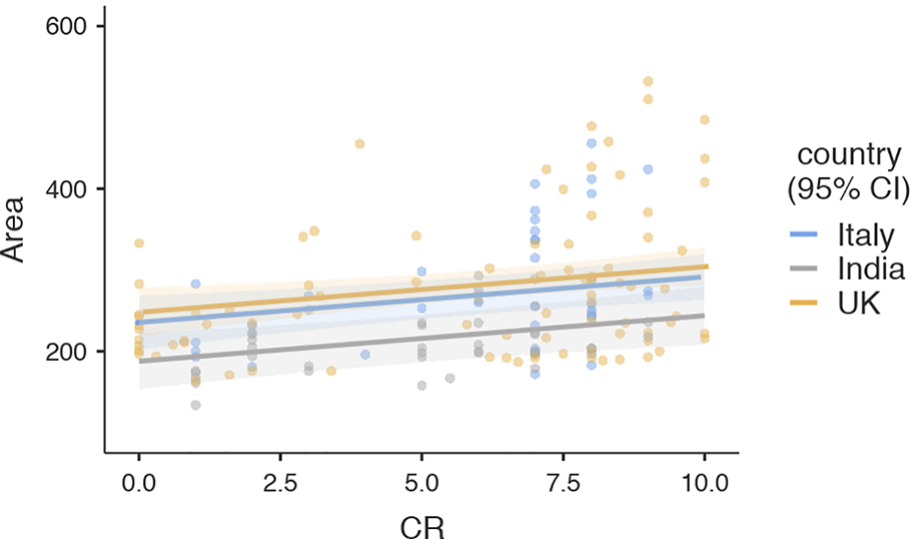
Scattergram illustrating the relationship between closeness rating and articulation space. Regression line and confidence intervals correspond to results of the hierarchical linear model presented in **Table 2** (Area: Articulation Space, CR: Closeness Rating).

**Figure 3:**
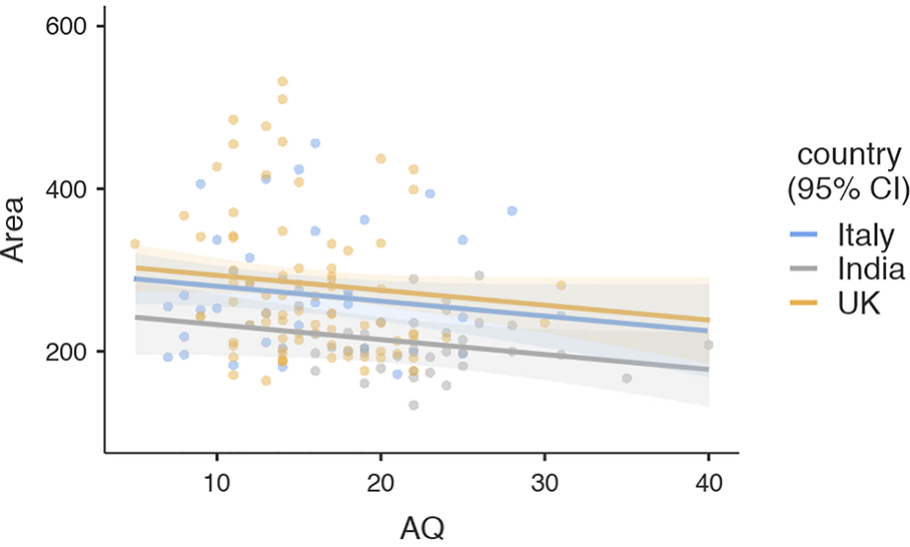
Scattergram illustrating the relationship between autism spectrum quotient and articulation space. Regression lines and confidence intervals correspond to results of the hierarchical linear model presented in **Table 2** (Area, Articulation Space; AQ, Autism Spectrum Quotient).

To visualize the relationship of CR with articulation space, a scattergram was plotted between closeness rating and articulation space (**Figure 2**). As indicated by the HLM, articulation space increased with greater interpersonal closeness. Similarly, the suggestive negative association with AQ was visualized using a scattergram, indicating that people with higher autistic traits used slightly less articulation space (**Figure 3**).

## Discussion

This study investigated the variability in articulation space during dyadic vocal communication in relation to two factors: interpersonal closeness of the interlocutors, as well as individual autistic traits. Articulation space was positively associated with interpersonal closeness, i.e., individuals used more articulatory gestures when they spoke to those who they rated high on closeness, compared to others who they rated low. A suggestive negative association between autistic traits and articulation space was noted.

The relationship between closeness rating and articulation space was found to be significant even after accounting for variation driven due to gender, age, and culture. Anecdotal accounts suggest that people use greater modulation of their voice during speaking to familiar others compared to strangers. This aspect of deliberate vocal control has been noted in other nonhuman primates and predates human speech (1). From a functional perspective, articulation space can arguably contain informative cues about group membership, and hence might subserve social bonding processes. Communication accommodation theory suggests that one of the affective motives of interpersonal vocal communication is to manage and regulate social distance (30–32). Increased closeness was associated with a greater articulation space, which suggests that (a) more information is being communicated with a closer other, through incorporation of greater non-verbal signals, and/or (b) individuals are more inhibited by social norms when talking with someone who they are not close to, and thus reduce the number/extent of their articulatory gestures. The current data set does not allow us to discriminate effectively between these two possibilities.

Beyond factors specific to the dyad, such as how close an individual felt toward another, the impact of individual variation in autism-related traits was measured. A weak negative relationship was observed between articulation space and autism-related traits. These results are in concordance with an earlier study conducted with toddlers with autism within a free play setting (24). While the focus of that study was on obtaining information about different articulatory features at different timescales, a reduced articulation space was also noted in toddlers with ASD. That autism is associated with atypical prosody (14, 33–37) is now well established. The characteristics of this atypical prosody however are less clear with one set of reports supporting “monotonic” speech, while other results reporting an increased pitch range in both using single word utterances as well as narratives (15, 34–36). The articulation space approach captures a wider set of acoustic features. The current results are consistent with previous studies on individuals with ASD which have shown that higher autistic traits are associated with reduced articulatory gestures. Further studies can focus on combining such articulation space analysis with studies in speech perception and hopefully establish the link between perception, speech motor skills, and speech production in autism.

It is worth considering a potential caveat with regard to the interpretation discussed above. The paradigm involved two individuals taking one turn each to speak to the other, without interruption. This design is therefore not a true conversation, which is marked by multiple turn-takings, and arguably greater vocal modulation. However, in light of the previous literature on audience effects in humans as well as other animals (38, 39), and the consistent effects of closeness on articulation space in all three samples, it is reasonable to assume that the participants did indeed modulate their voice in response to who they were speaking to. It should be noted though, that several potential sources of individual and dyadic variation were not formally investigated in this current study. Gender is one such variable, which accounts for significant differences in vocal modulation in the interpersonal context. Individuals might differ in how they speak to a member of the opposite sex/gender compared to one of the same sex/gender. While we found a significant main effect of gender (male pairs were associated with greater articulation space compared to female pairs), there were no mixed gender pairs in this study to systematically examine the impact of gender on articulation space. Variation in social contexts (e.g. if a conversation is happening between friends at a pub vs at the workplace) can also potentially lead to differences in articulation space. These sources of variation need to be explored in future studies.

In sum, this study found the impact of closeness on vocal modulation in interpersonal communication, demonstrating that a greater closeness was associated with more modulation, across different cultural and language settings. This study also found that autism-related traits showed a weak association with the extent of such vocal modulation. Future studies should extend these paradigms to include individuals with clinical deficits in social communicative abilities, such as those with ASD.

## Data Availability Statement

All data and code are available at: https://tinyurl.com/sssc2020.

## Ethics Statement

All protocols were approved by the Research Ethics Committees of the University of Reading, Università degli studi di Milano Bicocca, and National Brain Research Centre India and written informed consent to participate was obtained from each participant after emphasizing that (a) all participants would remain anonymous and data would be kept strictly confidential and (b) participants were free to withdraw their consent at any time with no unfavorable consequences.

## Author Contributions

The study was designed by NS and BC, the data were collected by OS under supervision from BC and NS, data analysis was conducted by TS and BC, and all authors contributed to the writing of the manuscript. All authors read and approved the final manuscript.

## Funding

BC was supported by the Leverhulme Trust (Grant No: PLP-2015-329), Medical Research Council UK (Grant No: MR/P023894/1), and SPARC UKIERI funds (Grant No: P1215) during this period of work. NS was supported by intramural funding from the National Brain Research Centre, India.

## Conflict of Interest

The authors declare that the research was conducted in the absence of any commercial or financial relationships that could be construed as a potential conflict of interest.

## Abbreviations

CR, closeness rating; AQ, autism spectrum quotient; SMS, speech modulation spectrum; ASD, autism spectrum disorders; UK, United Kingdom; PCM, pulse-code modulation; NAQ, normalized amplitude quotient.
